# Adverse Outcomes after Non-Cardiac Surgeries in Patients with Heart Failure: A Propensity-Score Matched Study

**DOI:** 10.3390/jcm10071501

**Published:** 2021-04-04

**Authors:** Po-Han Lo, Chuen-Chau Chang, Chun-Chieh Yeh, Li-Chin Sung, Yih-Giun Cherng, Ta-Liang Chen, Chien-Chang Liao

**Affiliations:** 1 Department of Anesthesiology, Shuang Ho Hospital, Taipei Medical University, New Taipei City 235, Taiwan; silver156m6@yahoo.com.tw (P.-H.L.); stainless@s.tmu.edu.tw (Y.-G.C.); 2 Department of Anesthesiology, School of Medicine, College of Medicine, Taipei Medical University, Taipei 110, Taiwan; nekota@tmu.edu.tw (C.-C.C.); tlc@tmu.edu.tw (T.-L.C.); 3 Department of Anesthesiology, Taipei Medical University Hospital, Taipei 110, Taiwan; 4 Anesthesiology and Health Policy Research Center, Taipei Medical University Hospital, Taipei 110, Taiwan; 5 Department of Surgery, China Medical University Hospital, Taichung 404, Taiwan; b8202034@gmail.com; 6 Department of Surgery, University of Illinois, Chicago, IL 60637, USA; 7 Division of Cardiology, Department of Internal Medicine, Shuang Ho Hospital, Taipei Medical University, New Taipei City 235, Taiwan; 10204@s.tmu.edu.tw; 8 Department of Anesthesiology, Wan Fang Hospital, Taipei Medical University, Taipei 116, Taiwan; 9 Research Center of Big Data and Meta-Analysis, Wan Fang Hospital, Taipei Medical University, Taipei 116, Taiwan; 10 Centers of Regional Anesthesia and Pain Medicine, Wan Fang Hospital, Taipei Medical University, Taipei 116, Taiwan; 11 School of Chinese Medicine, College of Chinese Medicine, China Medical University, Taichung 404, Taiwan

**Keywords:** adverse outcomes, complications, heart failure, mortality, noncardiac surgeries

## Abstract

The impact of heart failure (HF) on postoperative outcomes is not completely understood. Our purpose is to investigate complications and mortality after noncardiac surgeries in people who had HF. In the analyses of research data of health insurance in, we identified 32,808 surgical patients with preoperative HF and 32,808 patients without HF undergoing noncardiac surgeries. We used a matching procedure with propensity score and considered basic characteristics, coexisting diseases, and information of index surgery between patients with and without HF. Adjusted odds ratios (ORs) and 95% confidence intervals (CIs) for complications and mortality after noncardiac surgeries in patients with HF were analyzed in multivariate logistic regressions. HF increased the risks of postoperative acute myocardial infarction (OR 2.51, 95% CI 1.99–3.18), pulmonary embolism (OR 2.46, 95% CI 1.73–3.50), acute renal failure (OR 1.97, 95% CI 1.76–2.21), intensive care (OR 1.93, 95% CI 1.85–2.01), and 30-day in-hospital mortality (OR 1.80, 95% CI 1.59–2.04). Preoperative emergency care, inpatient care, and injections of diuretics and cardiac stimulants due to heart failure were also associated with mortality after surgery. Patients with HF had increased complications and mortality after noncardiac surgeries compared with those without HF. The surgical care team may consider revising the protocols for perioperative care in patients with HF.

## 1. Introduction

Heart failure (HF) is one of the leading causes of morbidity and mortality worldwide [1]. With the increasing prevalence and incidence of HF, it was estimated that 6.5 million adults had HF in the United States [2]. The total cost of HF was 30.7 billion US dollars in the United States in 2012, and it is expected to reach 69.7 billion dollars by 2030 [3]. The importance of HF on societal and economic burden needs to be valued.

Large population cohort studies on the outcomes after noncardiac surgeries in HF patients are scant, and higher mortality and the risk of readmission within 30 days in HF patients have been documented [4,5,6]. Furthermore, in a recent study, patients with HF were found to have a higher mortality rate even after 90 postoperative days [7]. However, there were study limitations in the previous studies included the lack of data on postoperative adverse events [4,5,6], a focus on the elderly patient group [4,5], no control group [6], no classification of HF severity [4,5,6], and no propensity-score matching procedures [4,5,6,7]. Furthermore, there are inconsistent findings about the higher postoperative 30-day mortality rate and readmission rate in HF patients [8]. The previous study showed that postoperative mortality increased in patients with HF but this association became insignificant after propensity-matching procedures [8]. Due to these inconclusive results, sophisticated and well-designed research is needed.

After applying a regression-adjusted propensity-score matching procedure, we conducted a surgical cohort study based on database of healthcare insurance. The purpose of this study is to investigate the influence of HF on outcomes after noncardiac surgery.

## 2. Methods

### 2.1. Data Source

Taiwan’s National Health Insurance program was implemented in March 1995. This study used research data of this insurance program that covers more than 99% of Taiwan’s population included 23 million residents. This research database included patients’ basic sociodemographic characteristics, information of medical services of ambulatory care and hospitalization (such as physicians’ prescriptions, medical expenditures, treatment procedures, and diagnosis.

According to the formal regulations from the Ministry of Health and Welfare, informed consent was not required because the information of research data was deidentified, and patient identifications were not available in this insurance database. However, this study was approved by Taipei Medical University (TMU-JIRB-201905042; TMU-JIRB-201902053).

### 2.2. Study Design

The surgical database included 3,000,000 adults who were aged 20 years and received inpatient surgeries in 2008–2013 in Taiwan. We identified 32,808 patients with HF who were aged 30 years and older, and each patient with HF was matched with one patient without HF who was selected by the propensity-score matching procedure. To select appropriate patients with and without HF for comparison, we considered baseline characteristics, including socioeconomics, medical conditions, recent experience of medical care, surgical procedure, mode of anesthesia in the matching procedure.

### 2.3. Measures and Definitions

In this study, we defined inpatient surgeries required general, epidural, or spinal anesthesia with hospitalization at least one day. The criterion ‘low income’ was based on the definition from the Ministry of Health and Welfare in Taiwan. The patients’ history of disease and medical care were identified within 24 months preoperatively. In this National Health Insurance system, we used physicians’ diagnoses and the International Classification of Diseases, Ninth Revision, Clinical Modification (ICD-9-CM) to identify heart failure (code 428) medical conditions and postoperative complications. History of disease and conditions that were determined from medical care for the previous 24 months before surgery included hypertension (code 401-405), ischemic heart disease (code 410-414), diabetes (code 250), mental disorders (code 290-319), chronic obstructive pulmonary disease (code 491, 492, 496), anemia (code 280-285), atrial fibrillation (code 427.3), chronic kidney disease, hyperlipidemia (code 272.0, 272.1, and 272.2), Parkinson’s disease (code 332), and liver cirrhosis (code 571.2, 571.5, 571.6). Renal dialysis was defined by administration codes (D8 and D9). We used ICD-9-CM codes and physician’s diagnosis to identify the characteristics of HF, such as rheumatic HF (code 398.91), malignant hypertensive HF (code 402.01, 404.01, 404.03), congestive HF (code 428.0), left HF (code 428.1), and unspecified HF (code 428.9).

The short-term mortality and complications after the index surgery within 30 days was considered the study’s outcomes included septicemia (codes 038 and 998.5), pneumonia (code 480-486), stroke (codes 430-438), urinary tract infection (code 599.0), acute renal failure (code 584), deep wound infection (code 958.3), pulmonary embolism (code 415), postoperative bleeding (codes 998.0, 998.1, and 998.2), and acute myocardial infarction (code 410). Consumption of medical resources were also considered in this study included intensive care, length of stay, and medical cost during the index surgical hospitalization.

### 2.4. Statistical Analyses

We used propensity-score matched pair analysis to determine associations between HF and postoperative outcomes. Using a non-parsimonious multiple logistic regression, we matched HF patients to non-HF patients by a greedy matching algorithm, and our purpose is to balance the distribution of covariates (potential confounding factors, such as age, gender, income status, and history of diseases) between people with HF and those who had no HF.

Baseline characteristics were presented as frequency with percentage for categorical variables and mean±SD for continuous variables. Chi-square tests for categorical variables and 2-sample t tests for continuous variables, as appropriate, were used to compare baseline characteristics of people with HF and those who had no HF. The multiple logistic regressions were used to calculate the adjusted odds ratios (ORs) and 95% confidence intervals (CIs) of postoperative complications and mortality associated with HF. The sensitivity analyses were also performed to investigate the risk of adverse outcomes after surgery in people with HF within subgroups. We used multiple linear regressions to calculate beta and *p*-value for medical expenditure and length of hospital associated with HF. In addition, the effects of characteristics of HF (such as rheumatic HF, malignant hypertensive HF, congestive HF, left HF, and unspecified HF) on the postoperative mortality were also analyzed by calculating adjusted ORs and 95% CIs.

## 3. Results

The basic information of the patients with HF and non-HF controls received surgeries are shown in Table 1 and Supplementary Materials Table S1. After propensity-score matching, the significant difference in demographic information, clinical medical conditions, types of surgery, and anesthesia were not detected between surgical patients with and without HF.

Compared with the non-HF group (Table 2), people with HF were more likely to have postoperative complications, including acute myocardial infarction (OR 2.51, 95% CI 1.99–3.18), pulmonary embolism (OR 2.46, 95% CI 1.73–3.50), acute renal failure (OR 1.97, 95% CI 1.76–2.21), pneumonia (OR 1.50, 95% CI 1.40–1.60), septicemia (OR 1.42, 95% CI 1.33–1.51), stroke (OR 1.30, 95% CI 1.21–1.39), and urinary tract infection (OR 1.19, 95% CI 1.13–1.26). We found increased risks of intensive care (OR 1.93, 95% CI 1.85–2.01) and mortality (OR 1.80, 95% CI 1.59–2.04) after surgery were in people with HF. Patients in HF group had longer lengths of stay (8.0 vs. 7.0 days, *p* < 0.0001) and higher expenditures (2701 vs. 2382 USD, *p* < 0.0001) than patients in non-HF group.

In the stratified analyses (Table 3 and Supplementary Materials Table S1), HF was associated with 30-day in-hospital mortality after surgery in women (OR 1.81, 95% CI 1.48–2.21), men (OR 1.79, 95% CI 1.53–2.10), and patients aged 30–44 years (OR 6.52, 95% CI 1.36–31.4), 45–54 years (OR 2.39, 95% CI 1.18–4.82), 55–64 years (OR 2.48, 95% CI 1.53–4.02), 65–74 years (OR 2.02, 95% CI 1.49–2.74), 75–79 years (OR 1.53, 95% CI 1.12–2.08), 80–84 years (OR 1.85, 95% CI 1.42–2.42), and ≥85 years (OR 1.58, 95% CI 1.28–1.97). The association between HF and 30-day in-hospital mortality after surgery was also significant within patient subgroups based on number of medical conditions (0, 1, 2, ≥3), number of hospitalizations (0, 1, 2, ≥3), number of emergency visits (0, 1, 2, ≥3), and types of anesthesia (general or regional). Supplementary Materials Table S2 showed the joint effects of age and HF on the risk of the 30-day in-hospital mortality after surgery. There was an interaction relationship between age and HF (*p* < 0.0001).

Compared with people had no HF (Table 4), patients in the HF group whose medical care for HF included emergency care (OR 2.14, 95% CI 1.81–2.54), inpatient care (OR 2.22, 95% CI 1.94–2.54), injections of diuretics (OR 2.04, 95% CI 1.77–2.35) and cardiac stimulants (OR 2.38, 95% CI 1.96–2.90) were more likely to have increased 30-day in-hospital mortality after surgery. In contrast, HF patients who had none the above treatment experiences did not have a significantly increased risk of 30-day in-hospital mortality after surgery (OR 0.95, 95% CI 0.75–1.22).

In the further analyses, we additional added Charlson Comorbidity Index, injections of diuretics, and injections of cardiac stimulant into the matching procedure with propensity score (Supplementary Materials Table S3). We found that HF was associated with postoperative complications and mortality (Supplementary Materials Table S4).

## 4. Discussion

Patients with HF who received noncardiac surgeries had a higher 30-day in-hospital mortality rate than those without HF in the current research, and the result is compatible with prior studies [4,5,6,7,8]. Our findings also indicated more postoperative complications in HF patients, including stroke, pulmonary embolism, pneumonia, septicemia, and urinary tract infection, acute myocardial infarction, and renal failure. HF patients were more likely to need intensive care and have longer hospital stays and high medical expenditures.

Our study found that the risk of pulmonary embolism after surgery was increased in patients had HF, and this information was not recognized in prior large population studies [4,5,6,7,8]. HF is well-documented as an independent risk factor for venous thromboembolism [9,10], and the pathophysiology may be due to low cardiac output, abnormalities of hemostasis, platelet dysfunction, and endothelial dysfunction in HF patients [11]. Central venous catheters and pacemaker leads, which may be frequently applied perioperatively in HF patients, also increase the risk of upper-extremity deep vein thrombosis [11]. Since hospitalized patients with previous surgery have the highest venous thromboembolism risk [12], medical staff should pay even more attention to perioperative HF patients and exploit appropriate prophylactic methods, such as early mobilization and pharmacologic and nonpharmacologic thromboprophylaxis.

It has been proposed that postoperative acute myocardial infarction and stroke share the same pathophysiology: acute thrombosis and atheromatous plaque rupture, which may be due to perioperative processes such as hemodynamic instability, cardiovascular stress, autonomic overstimulation, and disseminated hypercoagulability [13]. With a regression-adjusted propensity-score matching procedure, this study found that HF patients had significantly higher acute myocardial infarction and stroke risk after noncardiac surgery, and that there may be a predisposition for abnormal blood flow, endothelial dysfunction, and abnormal blood constituents (Virchow’s triad) in HF patients [14,15,16,17,18]. In addition to acute thrombosis and atheromatous plaque rupture, prolonged ST-depression–type ischemia may contribute to perioperative myocardial infarction [19], and watershed infarction may also result in perioperative stroke [20]; there might also be unstable hemodynamics and inadequate organ perfusion in perioperative HF patients due to sympathetic nervous system and renin-angiotensin-aldosterone system activation, medical therapeutic effects, and decreased cardiovascular revserve [21].

In this study, more postoperative infections were noted in patients with HF. It was suggested that HF impacts the immune system, which may increase the risk of infection because of the decreased cardiovascular reserve to supply sufficient blood to fight infection [22]; this might be the reason why HF patients had significantly higher postoperative pneumonia, septicemia, and urinary tract infection rates in this study. Furthermore, in this study, HF patients had a higher postoperative intensive care unit stay rate, which might also explain the higher infection rate in the HF patients. It was well-documented that intensive care unit-acquired infections such as pneumonia, urinary tract infection, and bloodstream infection were common in patients who had a stay in the intensive care unit [23,24,25], which is quite compatible with the findings in this study.

The pathophysiology of perioperative acute renal injury is multifactorial, including hemodynamic-mediated, damage-associated and molecular pattern-induced inflammation, urinary tract obstruction, and nephrotoxic medications such as antibiotics, nonsteroidal anti-inflammatory drugs, angiotensin-converting enzyme inhibitors, and angiotensin II receptor blockers [26]. With a propensity-score matching procedure, we identified that HF patients had a higher postoperative acute renal failure risk, which might be attributed to a hemodynamic-mediated mechanism. In a prospective observational study, patients with postoperative acute renal failure were more likely to receive vasopressor boluses or infusions during the operation, and both were independent predictors of postoperative acute renal failure [27]. Vasopressors would be used to prevent inadequate renal perfusion under a condition of hypotension, which is common in perioperative HF patients [28,29]. In addition to hemodynamic instability, in this study, HF patients had a higher postoperative infection rate, which might also contribute to the increased acute renal failure rate [30]. It is well-known that in sepsis patients, bacteremia and endotoxemia induce nitric oxide-mediated arterial vasodilatation, followed by increased sympathetic tone, renin-angiotensin-aldosterone system activation, and increased arginine vasopressin, which results in renal vasoconstriction and predisposes sepsis patients to acute renal failure [31].

The impact of HF severity on the postoperative 30-day mortality rate was difficult to validate because of the unavailable left ventricular ejection fraction records in the current research data. We tried to differentiate the severity of HF by previous medical records, including emergency care, inpatient care, injections of diuretics, and injections of cardiac stimulants in patients with a HF diagnosis. All 4 subgroups of HF patients had an even higher risk of 30-day mortality than the general HF patients, and those who received cardiac stimulant injections were at the highest risk. Furthermore, HF patients not belonging to the 4 subgroups do not have a higher 30-day mortality risk than those without HF, which accordingly might consolidate our stratification of HF severity. These findings possibly verified the impact of HF severity on postoperative outcomes [32].

The strengths of this study included large sample size, propensity score matching, multiple regression adjustment for potential confounding factors, and the comprehensive assessment of postoperative complications and mortality. However, our study has some limitations. First, clinical scores, such as severity of HF (the New York Heart Association Functional Classification), and echocardiographic categories, such as heart failure with midrange ejection fraction, heart failure with preserved ejection fraction, and heart failure with reduced ejection fraction, were not available. The detailed information on the results of examinations, causes of HF, and the related severity could not be available in the research data of Taiwan’s National Health Insurance. Second, prior studies utilized left ventricular ejection fraction to classify HF severity [7,8]; however, diastolic dysfunction might also be a potential risk factor in mild asymptomatic left ventricular systolic dysfunction patients [33]. In this study, diastolic dysfunction records were not included. Third, there might be undiagnosed HF patients in the non-HF group, and the impact of HF on postoperative outcome would be underestimated. Fourth, detailed sociodemographic information, cigarette smoking, alcohol drinking, and physical exercise, and biochemical laboratory data were not available. Finally, there is a possibility of potential confounding in our study, even though we used matching analyses by propensity score.

## 5. Conclusions

In conclusion, we found increased risks of complications and mortality in patients with HF than in those without HF. Comprehensive preoperative assessment and sophisticated perioperative care are recommended. Further well-designed prospective studies are needed to clarify the impact of HF on postoperative outcomes.

## Figures and Tables

**Table 1:** Characteristics of surgical patients with and without heart failure.

	No HF (*n* = 32,808)		HF (*n* = 32,808)		*p*-Value
Sex	*n*	(%)	*n*	(%)	1.0000
Female	16,543	(50.4)	16,543	(50.4)	
Male	16,265	(49.6)	16,265	(49.6)	
Age, years					1.0000
30–44	631	(1.9)	631	(1.9)	
45–54	1624	(5.0)	1624	(5.0)	
55–64	3918	(11.9)	3918	(11.9)	
65–74	8543	(26.0)	8543	(26.0)	
75–79	6070	(18.5)	6070	(18.5)	
80–84	6363	(19.4)	6363	(19.4)	
≥85	5659	(17.3)	5659	(17.3)	
Low income	253	(0.8)	253	(0.8)	1.0000
Types of surgery	*n*	(%)	*n*	(%)	1.0000
Skin	344	(1.1)	344	(1.1)	
Musculoskeletal	13,347	(40.7)	13,347	(40.7)	
Respiratory	1026	(3.1)	1026	(3.1)	
Digestive	8583	(26.2)	8583	(26.2)	
Kidney, ureter, bladder	2533	(7.7)	2533	(7.7)	
Neurosurgery	4129	(12.6)	4129	(12.6)	
Eye	206	(0.6)	206	(0.6)	
Others	2640	(8.1)	2640	(8.1)	
Types of anesthesia					1.0000
General	22,380	(68.2)	22,380	(68.2)	
Regional	10,428	(31.8)	10,428	(31.8)	
Medical conditions *					
Hypertension	14,049	(42.8)	14,049	(42.8)	1.0000
Ischemic heart disease	7022	(21.4)	7022	(21.4)	1.0000
Diabetes	6697	(20.4)	6697	(20.4)	1.0000
Mental disorders	6358	(19.4)	6358	(19.4)	1.0000
COPD	5148	(15.7)	5148	(15.7)	1.0000
Anemia	2835	(8.6)	2835	(8.6)	1.0000
Chronic kidney disease	1054	(3.2)	1054	(3.2)	1.0000
Hyperlipidemia	999	(3.0)	999	(3.0)	1.0000
Renal dialysis	560	(1.7)	560	(1.7)	1.0000
Parkinson’s disease	613	(1.9)	613	(1.9)	1.0000
Liver cirrhosis	415	(1.3)	415	(1.3)	1.0000
Atrial fibrillation	268	(0.8)	268	(0.8)	1.0000
Number of hospitalizations *					1.0000
0	14,858	(45.3)	14,858	(45.3)	
1	9593	(29.2)	9593	(29.2)	
2	4048	(12.3)	4048	(12.3)	
≥3	4309	(13.1)	4309	(13.1)	
Number of emergency visits *					1.0000
0	14,038	(42.8)	14,038	(42.8)	
1	8286	(25.3)	8286	(25.3)	
2	4135	(12.6)	4135	(12.6)	
≥3	6349	(19.4)	6349	(19.4)	

COPD, chronic obstructive pulmonary disease; HF, heart failure. * Within preoperative 24 months.

**Table 2:** Postoperative complications, mortality, and medical consumptions in patients with heart failure.

	No HF (*n* = 32,808)		HF (*n* = 32,808)		Risk of Outcomes	
Postoperative Outcomes	Events	%	Event	%	OR	(95% CI) *
30-day in-hospital mortality	400	1.2	705	2.2	1.80	(1.59–2.04)
Postoperative complications						
Pulmonary embolism	44	0.1	108	0.3	2.46	(1.73–3.50)
Acute myocardial infarction	99	0.3	247	0.8	2.51	(1.99–3.18)
Acute renal failure	451	1.4	871	2.7	1.97	(1.76–2.21)
Pneumonia	1540	4.7	2213	6.8	1.50	(1.40–1.60)
Septicemia	1983	6.0	2700	8.2	1.42	(1.33–1.51)
Stroke	1650	5.0	2090	6.4	1.30	(1.21–1.39)
Urinary tract infection	2567	7.8	2995	9.1	1.19	(1.13–1.26)
Postoperative bleeding	182	0.6	203	0.6	1.12	(0.91–1.37)
Deep wound infection	150	0.5	180	0.6	1.20	(0.97–1.49)
ICU stay	5706	17.4	8617	26.3	1.93	(1.85–2.01)
Medical expenditure, USD ^†^	2382 (3096)		2701 (3055)		*p* < 0.0001	
Length of hospital stay, days ^†^	7.0 (8.0)		8.0 (11.0)		*p* < 0.0001	

CI, confidence interval; HF, heart failure; OR, odds ratio; ICU, intensive care unit. * Adjusted for all covariates listed in Table 1. ^†^ Calculated by median (interquartile range) in the Kruskal-Wallis test; medical expenditure, USD: beta = 683, *p* < 0.0001; Length of hospital stay: beta = 1.7, *p* < 0.0001.

**Table 3:** The stratified analysis for mortality after noncardiac surgery associated with heart failure.

				30-Day in-Hospital Mortality			
		*n*		Deaths	Mortality, %	OR	(95% CI) *
Female	No HF	16,543	155		0.9	1.00	(reference)
	HF	16,543	275		1.7	1.81	(1.48–2.21)
Male	No HF	16,265	245		1.5	1.00	(reference)
	HF	16,265	430		2.6	1.79	(1.53–2.10)
Age 30–44 years	No HF	631	2		0.3	1.00	(reference)
	HF	631	11		1.7	6.52	(1.36–31.4)
Age 45–54 years	No HF	1624	12		0.7	1.00	(reference)
	HF	1624	27		1.7	2.39	(1.18–4.82)
Age 55–64 years	No HF	3918	24		0.6	1.00	(reference)
	HF	3918	58		1.5	2.48	(1.53–4.02)
Age 65–74 years	No HF	8543	64		0.8	1.00	(reference)
	HF	8543	127		1.5	2.02	(1.49–2.74)
Age 75–79 years	No HF	6070	69		1.1	1.00	(reference)
	HF	6070	104		1.7	1.53	(1.12–2.08)
Age 80–84 years	No HF	6363	87		1.4	1.00	(reference)
	HF	6363	158		2.5	1.85	(1.42–2.42)
Age ≥ 85 years	No HF	5659	142		2.5	1.00	(reference)
	HF	5659	220		3.9	1.58	(1.28–1.97)
0 medical condition	No HF	6378	62		1.0	1.00	(reference)
	HF	6378	94		1.5	1.54	(1.11–2.13)
1 medical condition	No HF	12,392	144		1.2	1.00	(reference)
	HF	12,392	260		2.1	1.84	(1.50–2.27)
2 medical conditions	No HF	9572	131		1.4	1.00	(reference)
	HF	9572	230		2.4	1.79	(1.44–2.23)
≥3 medical conditions	No HF	4466	63		1.4	1.00	(reference)
	HF	4466	121		2.7	1.98	(1.45–2.70)
Musculoskeletal surgery	No HF	13,347	90		0.7	1.00	(reference)
	HF	13,347	167		1.3	1.88	(1.45–2.44)
Respiratory surgery	No HF	1026	9		0.9	1.00	(reference)
	HF	1026	16		1.6	1.81	(0.79–4.13)
Digestive surgery	No HF	8583	188		2.2	1.00	(reference)
	HF	8583	328		3.8	1.79	(1.49–2.15)
Kidney, ureter, bladder	No HF	2533	20		0.8	1.00	(reference)
	HF	2533	36		1.4	1.83	(1.05–3.18)
Neurosurgery	No HF	4129	82		2.0	1.00	(reference)
	HF	4129	134		3.3	1.67	(1.26–2.20)

CI, confidence interval; HF, heart failure; OR, odds ratio. * Adjusted for all covariates listed in Table 1.

**Table 4:** The mortality after noncardiac surgery in patients with characteristics of heart failure.

		30-Day in-Hospital Mortality			
	*n*	Deaths	Mortality, %	OR	(95% CI) *
Control group (No HF)	32,808	400	1.2	1.00	(reference)
HF patients with					
Rheumatic heart failure	109	2	1.8	1.54	(0.38–6.34)
Malignant hypertensive heart failure	207	5	2.4	1.86	(0.75–4.59)
Congestive heart failure	26,920	622	2.3	1.87	(1.65–2.12)
Left heart failure	825	17	2.1	1.46	(0.89–2.40)
Unspecified heart failure	10,352	221	2.1	1.80	(1.52–2.13)
Have no any of following treatments	10,158	85	0.8	0.95	(0.75–1.22)
Emergency care	7113	220	3.1	2.14	(1.81–2.54)
Inpatient care	17,831	538	3.0	2.22	(1.94–2.54)
Injections of diuretics	14,897	438	2.9	2.04	(1.77–2.35)
Injections of cardiac stimulant	4231	156	3.7	2.38	(1.96–2.90)

CI, confidence interval; HF, heart failure; OR, odds ratio. * Adjusted for all covariates listed in Table 1.

## Data Availability

The data underlying this study is from the Health and Welfare Data Science Center. Interested researchers can obtain the data through formal application to the Health and Welfare Data Science Center, Department of Statistics, Ministry of Health and Welfare, Taiwan (http://dep.mohw.gov.tw/DOS/np-2497-113.html accessed on 3 April 2021). Under the regulations from the Health and Welfare Data Science Center, we have made the formal application (included application documents, study proposals, and ethics approval of the institutional review board) of the current insurance data. The authors of the present study had no special access privileges in accessing the data which other interested researchers would not have.

## Supplementary Materials

The following are available online at https://www.mdpi.com/article/10.3390/jcm10071501/s1, Table S1: The stratified analysis by medical visits, anesthesia, and history of disease for the association between heart failure and postoperative mortality, Table S2: The joint effects of age and heart failure on the risk of 30-day in-hospital mortality after major surgeries, Table S3: Characteristics of surgical patients with and without heart failure, Table S4: Postoperative complications, mortality, and medical consumptions in patients with heart failure.
