# Supplementary material for: Adverse Outcomes after Non-Cardiac Surgeries in Patients with Heart Failure: A Propensity-Score Matched Study
Source: J Clin Med. 2021 Apr 4;10(7):1501. doi: 10.3390/jcm10071501 (PMC8038504; doi:10.3390/jcm10071501)
Supplement: Supplementary file 1 [file jcm-10-01501-s001.pdf]

Table S1. The stratified analysis by medical visits, anesthesia, and history of disease for the association between heart failure and postoperative mortality

		n	30-day in-hospital mortality			
			Deaths	Mortality, %	OR	(95% CI)*
0 hospitalization	No HF	14858	114	0.8	1.00	(reference)
	HF	14858	221	1.5	1.98	(1.57-2.49)
1 hospitalization	No HF	9593	101	1.1	1.00	(reference)
	HF	9593	219	2.3	2.23	(1.76-2.83)
2 hospitalizations	No HF	4048	60	1.5	1.00	(reference)
	HF	4048	96	2.4	1.63	(1.17-2.26)
≥3 hospitalizations	No HF	4309	125	2.9	1.00	(reference)
	HF	4309	169	3.9	1.37	(1.08-1.74)
0 emergency visit	No HF	14038	107	0.8	1.00	(reference)
	HF	14038	211	1.5	2.01	(1.59-2.55)
1 emergency visit	No HF	8286	86	1.0	1.00	(reference)
	HF	8286	179	2.2	2.13	(1.64-2.76)
2 emergency visits	No HF	4135	52	1.3	1.00	(reference)
	HF	4135	112	2.7	2.22	(1.59-3.10)
≥3 emergency visits	No HF	6349	155	2.4	1.00	(reference)
	HF	6349	203	3.2	1.33	(1.07-1.64)
Regional anesthesia	No HF	10428	63	0.6	1.00	(reference)
	HF	10428	104	1.0	1.67	(1.22-2.29)
General anesthesia	No HF	22380	337	1.5	1.00	(reference)
	HF	22380	601	2.7	1.83	(1.59-2.09)
Hypertension	No HF	14049	134	1.0	1.00	(reference)
	HF	14049	290	2.1	2.22	(1.80-2.73)
Ischemic heart disease	No HF	7022	87	1.2	1.00	(reference)
	HF	7022	156	2.2	1.84	(1.41-2.40)
Diabetes	No HF	6697	70	1.1	1.00	(reference)
	HF	6697	143	2.1	2.10	(1.57-2.80)
Mental disorders	No HF	6358	94	1.5	1.00	(reference)
	HF	6358	171	2.7	1.87	(1.45-2.42)
COPD	No HF	5148	105	2.0	1.00	(reference)
	HF	5148	151	2.9	1.46	(1.13-1.89)
Anemia	No HF	2835	64	2.3	1.00	(reference)
	HF	2835	103	3.6	1.65	(1.20-2.28)
Atrial fibrillation	No HF	268	2	0.8	1.00	(reference)
	HF	268	10	3.7	5.85	(1.21-28.1)
Chronic kidney disease	No HF	1054	21	2.0	1.00	(reference)
	HF	1054	43	4.1	2.16	(1.26-3.69)
Hyperlipidemia	No HF	999	2	0.2	1.00	(reference)
	HF	999	2	0.2	1.00	(0.07-15.2)
Renal dialysis	No HF	560	13	2.3	1.00	(reference)
	HF	560	28	5.0	1.78	(1.57-2.03)
Parkinson's disease	No HF	613	9	1.5	1.00	(reference)
	HF	613	11	1.8	1.81	(1.60-2.06)
Liver cirrhosis	No HF	415	9	2.2	1.00	(reference)
	HF	415	14	3.4	1.68	(0.68-4.17)

CI, confidence interval; HF, heart failure; OR, odds ratio.

*Adjusted for all covariates listed in Table 1.

Table S2. The joint effects of age and heart failure on the risk of 30-day in-hospital mortality after major surgeries

			30-day in-hospital mortality			
		n	Deaths	Mortality, %	OR	(95% CI)*
Age 30-44 years	No HF	631	2	0.3	1.00	(reference)
	HF	631	11	1.7	5.65	(1.24-25.7)
Age 45-54 years	No HF	1624	12	0.7	1.97	(0.44-8.87)
	HF	1624	27	1.7	4.53	(1.07-19.2)
Age 55-64 years	No HF	3918	24	0.6	1.64	(0.39-6.98)
	HF	3918	58	1.5	4.03	(0.98-16.6)
Age 65-74 years	No HF	8543	64	0.8	2.16	(0.52-8.87)
	HF	8543	127	1.5	4.34	(1.07-17.7)
Age 75-79 years	No HF	6070	69	1.1	3.37	(0.82-13.8)
	HF	6070	104	1.7	5.13	(1.26-20.9)
Age 80-84 years	No HF	6363	87	1.4	3.94	(0.96-16.1)
	HF	6363	158	2.5	7.30	(1.80-29.7)
Age ≥85 years	No HF	5659	142	2.5	7.89	(1.94-32.1)
	HF	5659	220	3.9	12.5	(3.09-50.8)

CI, confidence interval; HF, heart failure; OR, odds ratio.

*Adjusted for all covariates listed in Table 1; there was an interaction relationship between age and HF ($p < 0.0001$).

Table S3. Characteristics of surgical patients with and without heart failure

	No HF (N = 17467)		HF (N = 17467)		p-value
Sex	n	(%)	n	(%)	1.0000
Female	9434	(54.0)	9434	(54.0)	
Male	8033	(46.0)	8033	(46.0)	
Age, years					1.0000
30-44	343	(2.0)	343	(2.0)	
45-54	917	(5.3)	917	(5.3)	
55-64	2143	(12.3)	2143	(12.3)	
65-74	4840	(27.7)	4840	(27.7)	
75-79	3196	(18.3)	3196	(18.3)	
80-84	3183	(18.2)	3183	(18.2)	
≥85	2845	(16.3)	2845	(16.3)	
Low income	99	(0.6)	99	(0.6)	1.0000
Medical conditions*					
Hypertension	7202	(41.2)	7202	(41.2)	1.0000
Ischemic heart disease	2689	(15.4)	2689	(15.4)	1.0000
Diabetes	2623	(15.0)	2623	(15.0)	1.0000
Mental disorders	2687	(15.4)	2687	(15.4)	1.0000
COPD	1721	(9.9)	1721	(9.9)	1.0000
Anemia	860	(4.9)	860	(4.9)	1.0000
Chronic kidney disease	262	(1.5)	262	(1.5)	1.0000
Hyperlipidemia	502	(2.9)	502	(2.9)	1.0000
Renal dialysis	134	(0.8)	134	(0.8)	1.0000
Parkinson’s disease	235	(1.4)	235	(1.4)	1.0000
Liver cirrhosis	126	(0.7)	126	(0.7)	1.0000
Atrial fibrillation	81	(0.5)	81	(0.5)	1.0000
Number of hospitalizations					1.0000
0	10117	(57.9)	10117	(57.9)	
1	4825	(27.6)	4825	(27.6)	
2	1276	(7.3)	1276	(7.3)	
≥3	1249	(7.2)	1249	(7.2)	
Number of emergency visits*					1.0000

0	9365	(53.6)	9365	(53.6)	
1	4303	(24.6)	4303	(24.6)	
2	1649	(9.4)	1649	(9.4)	
≥3	2150	(12.3)	2150	(12.3)	
CCI score					1.0000
1	9875	(56.5)	9875	(56.5)	
2	3886	(22.3)	3886	(22.3)	
3	1955	(11.2)	1955	(11.2)	
≥4	1751	(10.0)	1751	(10.0)	
Types of surgery					1.0000
Skin	138	(0.8)	138	(0.8)	
Musculoskeletal	7490	(42.9)	7490	(42.9)	
Respiratory	400	(2.3)	400	(2.3)	
Digestive	4610	(26.4)	4610	(26.4)	
Kidney, ureter, bladder	1080	(6.2)	1080	(6.2)	
Neurosurgery	2226	(12.7)	2226	(12.7)	
Eye	94	(0.5)	94	(0.5)	
Others	1429	(8.2)	1429	(8.2)	
Types of anesthesia					1.0000
General	11806	(67.6)	11806	(67.6)	
Regional	5661	(32.4)	5661	(32.4)	
Injections of diuretics					1.0000
No	12975	(74.3)	12975	(74.3)	
Yes	4492	(25.7)	4492	(25.7)	
Injections of cardiac stimulant					1.0000
No	16968	(97.1)	16968	(97.1)	
Yes	499	(2.9)	499	(2.9)	

COPD, chronic obstructive pulmonary disease; HF, heart failure.

Table S4. Postoperative complications, mortality, and medical consumptions in patients with heart failure

	No HF (N=17467)		HF (N=17467)		Risk of outcomes	
Postoperative outcomes	Events	%	Event	%	OR	(95% CI)*
30-day in-hospital mortality	180	1.0	304	1.7	1.72	(1.43-2.07)
Postoperative complications						
Pulmonary embolism	22	0.1	52	0.3	2.37	(1.44-3.91)
Acute myocardial infarction	43	0.3	101	0.6	2.37	(1.65-3.39)
Acute renal failure	227	1.3	408	2.3	1.83	(1.55-2.16)
Pneumonia	717	4.1	959	5.5	1.38	(1.25-1.53)
Septicemia	921	5.3	1204	6.9	1.35	(1.23-1.48)
Stroke	860	4.9	988	5.7	1.17	(1.06-1.29)
Urinary tract infection	1237	7.1	1433	8.2	1.18	(1.09-1.28)
Postoperative bleeding	90	0.5	98	0.6	1.09	(0.82-1.45)
Deep wound infection	86	0.5	85	0.5	0.99	(0.73-1.34)
ICU stay	2617	15.0	4035	23.1	1.95	(1.83-2.07)
Medical expenditure, USD†	3433±3843		3931±4559		p<0.0001	
Length of hospital stay, days†	10.0±11.2		11.2±12.6		p<0.0001	

CI, confidence interval; HF, heart failure; OR, odds ratio.

*Adjusted for all covariates listed in Table 1-1.

†Mean±SD; Medical expenditure, USD: beta=637, p<0.0001; Length of hospital stay: beta=1.6, p<0.0001
